# Meeting proceedings of the 43rd Indian Association for Cancer Research (IACR)

**DOI:** 10.1242/bio.061613

**Published:** 2024-08-14

**Authors:** Ajay J. Malik, Radhika Malaviya

**Affiliations:** Department of Biology, Indian Institute of Science Education and Research, Dr Homi Bhabha Road, Pune, Maharashtra 411008, India

**Keywords:** Cancer biology, IACR, Alternate careers, Annual meeting, Therapeutic modalities and outcomes, Research opportunities, Fundamental and translational research

## Abstract

The 43rd Annual Conference of the Indian Association of Cancer Research (IACR) was held between 19th and 22nd January 2024 at the Indian Institute of Education and Research (IISER), Pune, India. Cancer is the second leading cause of death globally; efforts have been made to understand and treat this deadly disease for several decades. The 43rd IACR, organised by Mayurika Lahiri, Kundan Sengupta, Nagaraj Balasubramanian, Mridula Nambiar, Krishanpal Karmodiya, and Siddhesh Kamat, highlighted recent advances in cancer research, with implications in therapeutics at the forefront of the discussions. The meeting proved to be a promising platform for cancer researchers ranging from graduate and postdoctoral students to subject experts in varied aspects of cancer biology to showcase their research, ideate with their peers, and form collaborations.

## Introduction

Cancer has continued to evade and evolve past the advancing treatment strategies and has become the second leading cause of mortality, accounting for 9.7 million deaths globally (WHO, 2022). This number is projected to increase at an alarming rate. A global unified effort is required to understand the basis and biology of cancer to curb the progression of this disease. At the 43rd IACR meeting, attendees shared their recent work in cancer research through talks and posters ranging from basic cancer biology to large-scale screens testing various patient treatment modalities.

## The conference

**Fig. 1.**
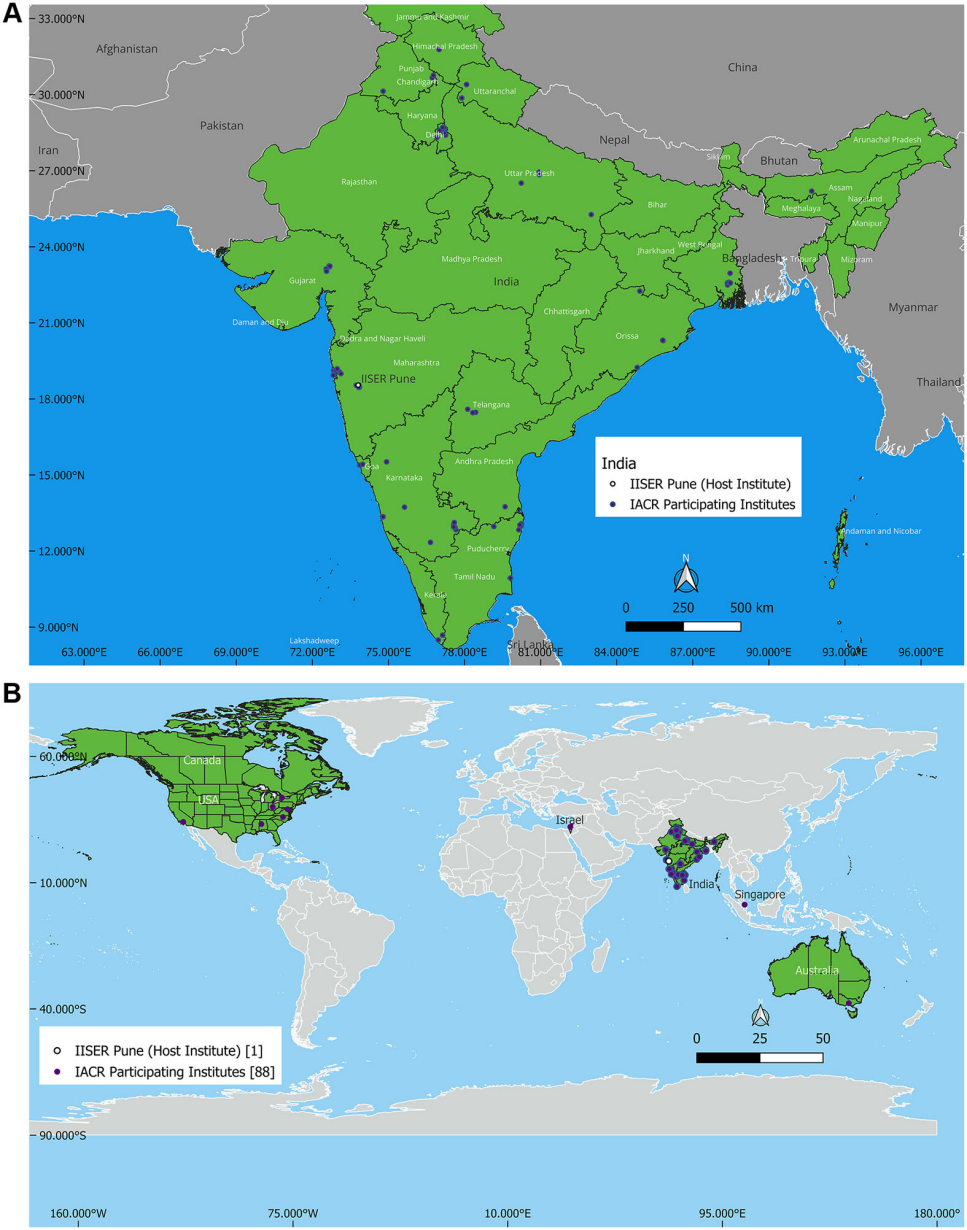
**Geographic locations of the 89 institutes participating in the IACR 2024 conference from (A) India-80; (B) outside India-9.** (Maps made using QGIS.org, 2024.)

**Fig. 2.**
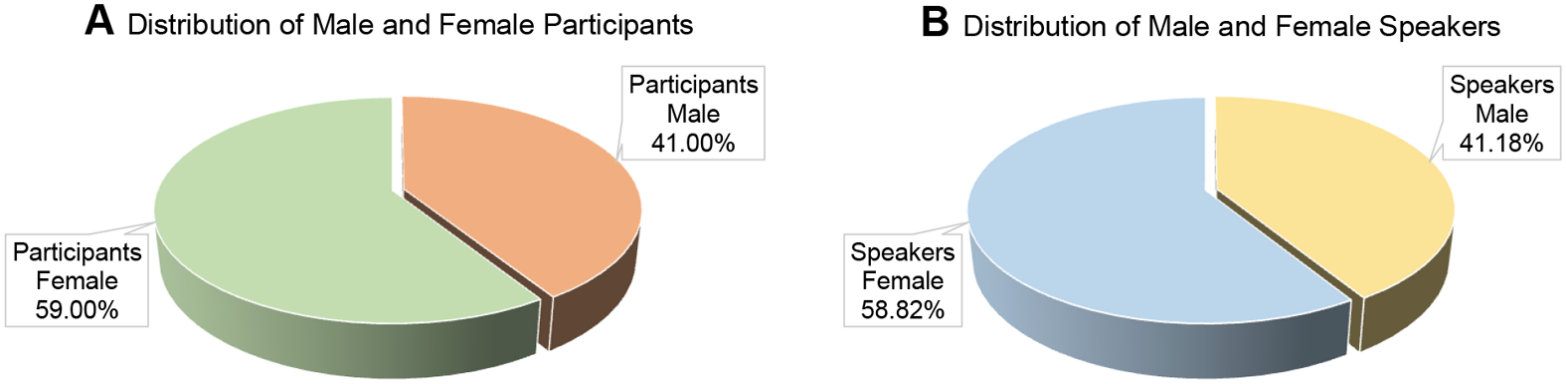
**Gender distribution.** (A) Participants; (B) speakers.

The 43rd IACR had 273 participants comprising of students, postdoctoral, and early to mid-career fellows from 80 research organisations nationwide and nine outside India (Fig. 1A,B). 34 of the 46 speakers were from within India. With an overall male-to-female ratio of 1:1.4, 59.00% of participants and 58.82% of the invited speakers were female (Fig. 2). The 4 days of the meeting were distributed into eight sessions, comprising the IACR presidential oration, five keynote lectures, the EMBO Young Investigator Lecture, 23 presentations, three early-mid career scientist talks, ten student flash-talks selected from the submitted student abstracts, three sponsor talks and a panel discussion on alternative careers. Along with the numerous talks, 150 poster presentations provided ample opportunities for speakers and students to discuss diverse aspects of cancer biology.

## Cancer initiation

Cancer initiation is a multi-step process where normal cells undergo irreversible changes and transform into malignant cells. A deeper understanding of these mechanisms helps inform a better understanding of the origin of cancer.

Nick Barker (IMCB, Singapore) shared his group's work on deciphering the gastric stem-cell niche to identify markers of gastric cancer stem-cells (CSC). They discovered Aquaporin 5 (Aqp5) as a marker of gastric CSCs. Aqp5 expression in cells can potentially give rise to invasive gastric cancer in mouse models (Tan et al., 2020). The group also showed that altered expression and localisation of Aqp5 could help understand the mechanism of gastric cancer initiation. Nishana Mayilaadumveettil (IISER Thiruvananthapuram) presented their work on the role of CTCF and CTCFL in genomic regulation. They found that CTCF and CTCFL have unique, combined transcription and chromatin organisation roles. The aberrant expression of CTCF and CTCFL might be responsible for the altered transcription of genes in cancer cells (Nishana et al., 2020), potentially contributing to carcinogenesis. Faseela EE (NCBS, Bengaluru), PhD student, linked CTCF/Cohesin binding sites to replication stress during S phase, possibly leading to genomic instability in cancers. In the early- to mid-career scientist talk, Abhijith Kuttanamkuzhi (Princess Margaret Cancer Center, Toronto) showed that cell-cycle checkpoint signalling differs between different lineages of breast epithelial cells. The differential signalling could provide new strategies for early interception in breast cancer (BC) (Kim et al., 2023). PhD students Amrapali Dutta (IISc, Bengaluru) discussed the biophysical basis of the interaction of cancer cell clusters with mammary epithelium that aids cell competition during cancer initiation, and Jugal Kishore Sahu (ILS, Bhubaneshwar) showed that POLD4 of human DNA polymerase-δ is required for genomic stability and is differentially expressed in various tumours. Its deletion could lead to increased replication stress.

## Cancer progression

Tumour progression is the next stage of tumour development characterised by increased growth and aggressiveness. Aberrant signalling cascades lead to increased cellular malignancy.

In the presidential oration, Sorab N. Dalal (ACTREC, Navi Mumbai) discussed the role of iron homeostasis in tumour progression and therapy resistance in colorectal cancer. His group demonstrated that lipocalin 2 (LCN2) is a potential therapeutic target in colorectal cancers. LCN2 plays a significant role in preventing ferroptosis in cancer cells (Chaudhary et al., 2021) and could promote invasion by increasing actin polymerisation (Choudhary et al., 2023), promoting focal adhesion formation. Shweta Tyagi (CDFD, Hyderabad) elucidated the role of mixed lineage leukemia (MLL) protein in cytoskeletal dynamics and tumorigenesis (Chinchole et al., 2022). They have shown that MLL regulates actin dynamics, stress fibre formation, spindle assembly, and cell shape. Their findings in mouse xenograft models also point toward the role of MLL in tumour progression. Medhavi Vishvakarma (IISc, Bengaluru) discussed the influence of spatio–temporal mechanics of cells on cancer initiation. Her group has developed tools to study the heterogeneity in local temporal oscillations occurring in actin expression and traction force in the cells. Traction force oscillations and differences in actin expression in cancer cell clusters could control cell competition during tumorigenesis.

Sharmila Bapat (NCCS, Pune) demonstrated a new approach to understanding the causes of transcriptome alterations in ovarian cancers. They found chimeric transcripts that might result in transcriptional plasticity, often observed at the protein level (Dixit and Bapat, 2023; Sridhar et al., 2023). Tuli Dey (SPPU, Pune) discussed tumoral stress management and how aggressive and non-aggressive spheroids react differently to various stresses *in vitro*. An exciting finding was that non-aggressive tumours displayed more resilience to stress (Gayan et al., 2024). Dey's talk underlines the importance of understanding such decision-making processes, providing insights into cancer's intelligence, leading to better combat strategies. Prasanna Venkatraman (ACTREC, Navi Mumbai) spoke about structurally and functionally perturbed proteasomes in her talk entitled ‘Deducing Cancer Proteasome Structure and Function’.

The student talks by Radhika Malaviya (IISER, Pune) explored the role and regulation of Golgi organisation in anchorage-independent lung cancers while SK Eashayan Tanbir (IICB, Kolkata) presented how VEGF affects lipid droplet dynamics and induces lipophagy in ovarian carcinoma.

## Invasion and metastasis

Tumour invasion involves the expansion of cancer cells into the nearby environment and is followed by metastasis. Cells become motile, undergo physical, cellular, and molecular changes navigating through the ECM to invade secondary sites.

Chandrima Das (SINP, Kolkata) discussed the role of chromatin readers in shaping breast tumour heterogeneity. Her group established UBR7 as a novel histone H2B monoubiquitin ligase that suppresses tumorigenesis and metastasis (Dasgupta et al., 2022) and suggested that UBR7 relates to Triple-negative BC (TNBCs) metastasis by remodelling the matrix via TGFβ signalling, thereby altering collagen content and lysyl oxidase activity. Radhika Nair (CHG, Bengaluru) focused on the molecular circuitry involved in spreading primary BC from the lesion site by intrinsic metabolic programs and changes in the tumour microenvironment. Their group identified MACC1 as a player in driving an aggressive disease phenotype in the primary tumour and was first to find a therapeutic targeting MACC1 (Thankamony et al., 2023). These data show the effectiveness of targeting heterogeneous tumour cells during therapy. Ramray Bhat (IISc, Bengaluru) focused on unique rheological properties imparted by matrisomal signatures and found that senescence-specific matrisomes enable carcinomatosis in ovarian cancers. He explained how glycation-driven aberrations in ECM contribute to the dicarbonyl stress-driven upregulation of cancer cell intravasation. ECM dynamics contribute to cancer progression and the difference in features between blastuloid spheroids and moruloid spheroids (Langthasa et al., 2021). Ramray emphasized the effect of Galectin 9 on the multimodal invasion into the stromal matrix (Pally et al., 2022). Snehasikta Swarnakar (IICB, Kolkata) discussed unpublished data focussing on the role of matrix metalloproteases (MMPs) and endopeptidases in epithelial ovarian cancer, found that miRNA34a expression inhibited *in vitro* tumorigenesis and inversely correlated with MMP2 activity while the stage of ectopic endometrial cancer tissue positively correlated with MMP2 activity. Furthermore, the oxytocin-MMP2 activity regulates angiogenesis.

During graduate student talks, Amulya Ichageri (SPPU, Pune) outlined a potential model for bone-like scaffolds to evaluate bone-specific migration of BC cells. Ritika Gupta (NCCS, Pune) presented work on the *in vitro* stratification of the syngeneic mouse model of ovarian cancer, to investigate differences in epithelial and mesenchymal phenotypes.

## Prognosis and potential biomarkers

Identifying biomarkers and understanding the disease prognosis could provide a better understanding of cancer and assist in formulating better strategies to treat the disease at early stages.

Lalit Sehgal (Ohio State University, Ohio) focused on developing new therapies to improve mantle cell lymphoma (MCL) outcomes. Lalit correlated FGFR1 levels with B-cell trajectory and cell state in MCL and its effect on EZH2 and cell cycle-related genes. Patients with CDKN1C downregulation and FGFR1 upregulation positively regulate E2F1-mediated transactivation of its target gene through cMYC/EZH2/CDKN1C axis, contributing to cell survival (Sircar et al., 2023). Sabarinathan Radhakrishnan (NCBS, Bengaluru) found that the immunoproteasome positively correlated with constitutive proteasomes in multiple tumour types. Tumours with high immunoproteasome expression have higher cytotoxic immune cell infiltration and are associated with the upregulation of INFγ and TNFα pathways in tumour cells (Kumar et al., 2023). Correlating immunoproteasome expression and infiltrating immune cell patterns could have implications for the prognosis of overall survival and response to immune checkpoint blocker treatments. Kartiki Desai (NIBMG, Kalyani) focused on the prognostic potential of circulating BC extracellular vesicles (EVs) in cancer progression and recurrence. The transcriptome of circulating EVs (cEVs) reveals that tumours secreting high JMJD6 showed lower ER expression and had poorer effects of Tamoxifen, leading to endocrine therapy resistance (Das et al., 2022). She reported the striking differences in RNA profiles of cEVs between those arising from cancer cells and those from normal healthy cells. Lekha Dinesh Kumar (CCMB, Hyderabad) explored the efficacy of miRNAs as potential biomarkers for invasive ductal carcinoma classification. They have evaluated miRNA signatures in patient samples of different types, grades, and stages of invasive ductal carcinoma. The discovery of novel miRNAs in patient samples and altered expression of some miRNAs compared to normal samples highlights the utility of this strategy in the classification and detection of cancers (Verma et al., 2024). Jyothi S. Prabhu (SJRI, Bengaluru) delved into evaluating androgen and glucocorticoid receptors as a clinical diagnostic/prognostic marker. Jyothi demonstrated that androgen and glucocorticoid receptors can provide significant prognostic information, especially in the TNBC cohort (Rajarajan et al., 2021). Lata Singh (AIIMS, New Delhi) discussed the regulation of ferroptosis in uveal melanoma. Her group has investigated the expression of ferroptotic genes and found that TFR1, GPX4, and SLCA11 might be implicated in uveal melanoma.

## Cancer therapy and translational potential

The numerous studies characterising the mode of action of cancer cells have contributed to formulating increasingly effective treatment strategies for patients. Several talks highlighted recent advances in the field, focusing on the benefits of personalising treatments for this heterogeneous disease.

In the EMBO Young Investigator Lecture, Uri Ben David (Tel Aviv University, Tel Aviv) focused on aspects of aneuploidy in cancer that could be used to develop treatment strategies. They found that breast-to-brain metastasis of BC is driven by Chr17p chromosome deletion and inactivated p53 (Laue et al., 2023 preprint). They also showed that KIF18A aberrations drive aneuploidy by skipping the spindle checkpoint (Cohen-Sharir et al., 2021). The dependence of human aneuploid cells on the RAF/MEK/ERK pathway to overcome DNA damage stress can be exploited to design treatment strategies that sensitise cells to DNA-damaging agents and PARP inhibitors. Lee Zou (Duke University, North Carolina) discussed his views of genome instability and mutation, a hallmark of cancer, as a driving force and vulnerability. Lee's lab is trying to exploit this using small-molecule ATR inhibitors. Lee demonstrated that ATR inhibition showed better synthetic lethality and increased anti-tumour immunity in cells with mismatch repair deficiency and higher infiltrating CD8+ cells in the tumour, improving prognosis in tumours exhibiting high replication stress (Wang et al., 2023). Bushra Ateeq (IIT, Kanpur) expressed interest in finding the recurrence of genetic alterations in Indian prostate cancer patients and the mechanism involved in SPINK1 upregulation leading to high Gleason scores and poor prognosis. They discovered that miRNA-338/421 activity could be restored using epigenetic drugs, resulting in reduced oncogenic potential caused by SPINK1 (Bhatia et al., 2019). Bushra discussed how targeting MALAT1 could augment PARP inhibitor sensitivity in metastatic castration-resistant prostate cancer patients, which provides a strong rationale for conducting clinical trials to investigate its efficacy in such patients (Yadav et al., 2023). Fayaz Malik (CSIR-IIIM, Srinagar) addressed two questions related to Trastuzumab resistance in HER2+ subtype: dysfunction of PTEN contributing to Trastuzumab prognosis and the expressional and functional significance of the AKT isoforms (AKT1/2/3) in stemness, invasion and cisplatin response in TNBC patient samples, mice and cellular models (Wadhwa et al., 2020). PTEN downregulation in HER2+ cells showed higher HERCEPTIN resistance and CSS-like properties, possibly caused by the inflammatory environment generated during resistance development. These findings have clinical implications for Trastuzumab treatment in HER2+ BC patients (Korkaya et al., 2012). Suresh Mathivanan (La Trobe Institute for Molecular Sciences, Melbourne) illustrated the role of EV in cancer progression and therapy. Milk-derived EVs appear to affect cancer progression variably at different stages. Suresh showed how the timing of EV administration is crucial due to the context-based effect of milk-derived EVs on cancer cell metastasis. Their studies also report EVs as a viable route for drug delivery in pre-clinical models (Samuel et al., 2021).

Anasuya Roychowdhury (IIT, Bhubaneshwar) addressed whether ATAD2 could be a potential target for therapy in gastrointestinal cancer. The work suggests that ATAD2 is a hypoxia-responsive gene regulated by HIF1α. Combined therapy targeting ATAD2, chemotherapy, and radiotherapy could effectively treat stomach and pancreatic cancers (Dutta et al., 2022). Divya P. Kumar (JSS Medical College, Mysuru) presented work on elucidating the molecular pathway involving AATF and evaluated AATF as a potential therapeutic target in steatohepatitis and hepatocellular carcinoma (Suresh et al., 2023). Mamoni Dash (ILS, Hyderabad) explored the utility of exosomes as nanoplatforms for targeted drug delivery in osteosarcoma, revealing that exosomes carry cargo that might have tumour suppressor mRNA. They have also developed strategies for surface engineering the exosomes to improve the targeting of exosomes to tumours.

Umar Khalid Khan (IIT, Kanpur) showed how attenuating DKC1 reduces oncogenic properties in colorectal cancer. Shivani Rajendrakumar Nandhap (HBNI, Mumbai) discussed the anticancer effects of Mitocurcumin, a modified version of curcumin that specifically targets mitochondria, which causes a redox imbalance.

## Therapy resistance

Despite the advances in cancer therapy, the plasticity and adaptability of cancer cells allow them to evade treatment. This necessitates better insights into the mechanism employed by tumours to evade therapy.

Sagar Sengupta (NIBMG, Kalyani) emphasised the functions, interactors, and chemotherapeutic potential of BLM, member of the RecQ helicase family. He demonstrated how BLM stimulates Rad54's ATPase and chromatin remodelling activity (Kaur et al., 2021). They screened FDA-approved small-molecule chemotherapeutics and explored the effects of BLM helicase inhibition on the mechanism of chemoresistance in colon cancer (Kaur et al., 2024). Anindya Dutta (University of Alabama, Alabama) showed that ASF1A, a histone chaperone coded by an intron of MCM9, works in tandem with 53BP1 to stop end-resection in HR repair and drive the repair pathway towards NHEJ (Lee et al., 2017). Anindya proposed that homozygous deletion of this gene, found in some prostate cancers may cause increased sensitivity to DNA-damaging agents. Moreover, germline variation of the GRB2 gene, upstream of the RAS-RAF-MAPK pathway, could influence somatic mutation of the transcription factor, Capicua, potentially leading to upregulation of the MAPK pathway, enhancing tumorigenesis. (Chatrath et al., 2021). Nirmalya Sen (Bose Institute, Kolkata) and his group focus on understanding the evolution of chemoresistance in TNBCs and found a protein that plays a significant role in developing chemoresistance to DNA-damaging agents. He further elucidated a lucrative target that may target these chemoresistant tumours. Shyamili Goutham (IISc, Bengaluru) discussed the trade-offs of acquiring chemoresistance compared to invasive and metastatic potential of cancer cells.

## Panel discussion on opportunities in alternate careers in science

The conference's final session focused on alternate careers in science, where three panellists described their professional journey. Manjiri Barke (OncoStem Diagnostics, Bengaluru) spoke about transitioning from a postdoctoral fellow to an entrepreneur. OncoStem provides prognostic services to patients with hormone-positive HER2-negative cancers conjointly with clinicians, allowing prediction of low and high-risk patients and providing a potential personalised treatment plan per the patient's requirements. Ipsa Jain (Shrishti Manipal Institute of Art, Design, and Technology, Bengaluru) discussed her work as a science illustrator in science communication and public engagement. She creates tools for science communication to connect the scientific community and the layman without expert intermediation. Mohor Sengupta (National Institutes of Health, Maryland) highlighted her role as a science policy analyst. She discussed her experience transitioning from a postdoctoral fellow working as a neuroscientist to a senior health science policy analyst for the NIH.

The following panel, discussion involved attendees and speaker interactions, where they further expanded on their work and individual experiences while transitioning out of academia. The conversation involved topics ranging from the challenges of switching from academia to the efforts to establish oneself in an alternate career.

## Poster presentation, student speaker, and essay-writing competition results

The poster presentation sessions provided a platform for many attendees to discuss and receive feedback on their work. Anurima Samanta (CNCI, Kolkata) received the ‘Rajanikant Shivprasad Baxi’ award, while Abhipsa Sinha (CSIR-CDRI, Lucknow) received the ‘Shri Rambhau Kulkarni’ award. The IACR 2024 poster awards were awarded to Sukanya Ghosh (CNCI, Kolkata), Antara Chakraborty (IISER, Pune), Ninja George (NCBS-TIFR, Bengaluru) and Neha Yadav (IISER, Berhampur). The student talks by Faseela EE and Umar Khalid Khan were selected for the ‘Sitaram Joglekar’ and ‘Mangala Bamne’ awards, respectively. Oindrila Ghosal (ACTREC, Navi Mumbai), Preeti Dahiya (University of Delhi, New Delhi), and Safiya Mehraj (CSIR-IIIM, Srinagar) won the IACR Annual Essay Writing Competition on the topic: ‘Translational research is not possible without basic research training’.
